# Prevalence, Characteristics, and Associated Risk Factors of Wrist Fractures in Americans Above 50: The Cross-Sectional NHANES Study

**DOI:** 10.3389/fendo.2022.800129

**Published:** 2022-04-25

**Authors:** Juncai Ye, Qiao Li, Jing Nie

**Affiliations:** ^1^ Department of Orthopedics, Zhejiang Hospital, Hangzhou, China; ^2^ Department of Orthopedics, The Second Clinical Medical College of Zhejiang Chinese Medical University, Hangzhou, China; ^3^ Department of Orthopedics, Traumatology and Orthopedics Hospital of Traditional Chinese Medicine of Xiaoshan District, Hangzhou, China

**Keywords:** wrist fracture, prevalence, cross-sectional study, NHANES, osteoporosis

## Abstract

**Summary:**

By analyzing data from NHANES, we aimed to evaluate the prevalence, characteristics, and associated factors of wrist fractures in Americans aged 50 and above.

**Introduction:**

Wrist fractures, whose prevalence increases with age, are one of the most common fractures in the United States. However, epidemiological studies on the prevalence of wrist fractures of certain ages were limited.

**Methods:**

The data of Americans aged 50 or above from 2013–2014 and 2017–2018 in NHANES were extracted and analyzed.

**Results:**

The prevalence of wrist fractures among Americans whose age was 50 or above was 12%, which was similar between men and women (men 12.8% vs. women 11.4%, *p* = 0.267). Among those who had experienced their first wrist fracture, 17.8% of the population experienced a second wrist fracture. The top two causes of the first wrist fracture were a fall from a standing height (56%) or a hard fall (34.8%). The prevalence of wrist fractures was higher in men than in women (13.7% versus 8.7%, *p* = 0.023) aged < 60, but higher in women than in men aged ≥ 60 (11.8% versus 14.3%, *p* = 0.007). Multivariate analysis showed that obesity, frequent drinking, current smoking, high serum phosphate level, non-Hispanic white women, and osteoporosis were independently associated with wrist fractures. Stratified by race, osteoporosis, frequent drinking, and high serum phosphate level were risk factors for wrist fractures in all races. As for Mexican Americans, non-Hispanic whites, and other races including multi-racial, current smoking was a risk factor of wrist factures. Furthermore, obesity was positively associated with wrist fractures in Mexican Americans, other Hispanics, and non-Hispanic whites.

**Conclusion:**

The prevalence of wrist fractures in Americans aged 50 and above was 12%. Falling from a standing height was the main cause of the first wrist fracture. Frequent drinking, current smoker, high serum phosphate level, osteoporosis, obesity, and non-Hispanic women were more likely to experience wrist fractures.

## Introduction

Wrist fractures are one of the most common fractures in the United States, accounting for about one-sixth of all fractures treated in emergency departments. The prevalence of wrist fractures increases with age (1–4). Studies have implied a significantly increased risk of recurrent wrist fractures within 7 years after the incidence of the initial wrist fracture, and risks of hip and vertebral fractures increased as well (5–7). Wrist fractures are usually followed by acute pain and swelling in the wrist and lead to dysfunction of wrist and even the quality of life if not being treated timely or appropriately (8, 9). There is also an increased risk of death following wrist fractures: within 5 years of the occurrence of distal fracture of the forearm, patients aged 65 to 74 have a death risk of 13.1%, while that of patients above 85 years old was 44.8% (10). It is predicted that by the year 2040, treatment cost for fractures would add up to $50 billion, which adds to the burden of the national healthcare system (11). In the U.S., the incidence rate of wrist fractures was three times higher than that of hip fractures. Therefore, even though the cost for hip fracture treatment per patient is higher, the overall treatment fee for wrist fractures surpasses that for hip and other fractures, which is why wrist fractures have drawn more attention from healthcare (7, 12). Therefore, it is high time that we design more studies to assess the prevalence, characteristics, and associated risk factors of wrist fracture, contributing to their prevention and treatment measures. However, epidemiological studies on incidence of wrist fractures were limited (5, 13). There has not been reports on the prevalence of wrist fractures yet. Therefore, this study aimed to comprehensively investigate the prevalence, characteristics, and related risk factors of wrist fracture among Americans aged 50 and above according to NHANES data in two year groups of 2013–2014 and 2017–2018. To the best of our knowledge, our study conducts the first research on patients with wrist fractures above 50 years of age based on data extracted from the NHANES database, which can enlighten the understanding for the disease as well as lending insight into prevention and improving prognosis.

## Methods

### Study Population

The National Health and Nutrition Examination Survey (NHANES) is a cross-sectional study of the American population collected every 2 years by the Center for Disease Control and Prevention (CDC), whose data are publicly available for use worldwide. NHANES was approved by the CDC for written informed consent from each participant. The data of 5,846 Americans aged 50 or above from 2013–2014 and 2017–2018 NHANES were extracted to estimate the prevalence of wrist fractures among the U.S. civilian population aged ≥ 50. All study subjects were examined physically and evaluated at medical centers. In this study, 4 subjects who had poor memory of their own past wrist fractures were excluded, while a total of 5,842 remaining subjects were included into the cohort, which contained 554 wrist fracture cases (male subjects, *n* = 239; female subjects, *n* = 315).

### Study Variables and Covariates

Self-reported conditions of individuals in the NHANES data were collected through methods of standardized questionnaires and medical evaluation at medical centers with professional assistance. To define diagnosis of current wrist fracture or wrist fracture history, participants were asked to recall whether they had previously been diagnosed with wrist fracture by a professional orthopedist. Answers included yes, no, refused, don’t know, and missing. Participants who refused to answer the questions, or whose answer is “I don’t know” or “missing” were excluded from the analysis. Details of questionnaires could be accessed online: https://wwwn.cdc.gov/Nchs/Nhanes/2013-2014/OSQ_H.htm and https://wwwn.cdc.gov/Nchs/Nhanes/2017-2018/OSQ_J.htm. In combination with the epidemiological characteristics of previous wrist fractures and NHANES data variables, we collected the following variables of Americans aged 50 and above: age, gender, number of fracture onsets, the specific age and reason of the first wrist fracture [reasons include (1) a fall from a standing height or less, for example, tripped, slipped, fell out of bed; (2) a hard fall, such as falling off a ladder or step stool, or down the stairs; and (3) a car accident or other severe trauma], race (Mexican Americans, other Hispanics, non-Hispanic whites, non-Hispanic blacks, and other race, including multi-racial), education (less than high school, high school or greater), marital status, smoking history, drinking history, obesity, hypertension, diabetes, osteoporosis, medication history of prednisone or cortisone, wrist fracture history of parents, and serum calcium and phosphorus level. Participants were defined as high blood pressure patients if they complied with at least one of the following conditions: (1) currently taking antihypertensive medications; (2) previously diagnosed by a physician; (3) four blood pressure measurements indicated a mean systolic blood pressure ≥ 130 mmHg; (4) diastolic blood pressure ≥ 80 mmHg (14). Obesity was defined by body mass index (BMI) ≥ 30 kg/m^2^ (15). Diabetes was defined if the participant complied with at least one of the following conditions: (1) diagnosed by a physician; (2) currently taking hypoglycemic drugs; (3) fasting blood glucose ≥ 130 mg/dl; (4) glycated hemoglobin ≥ 6.5% (16). Kidney conditions were estimated based on the glomerular filtration rate in serum creatinine: eGFR (ml/min/1.73 m^2^) = 175 × standardized Scr^−1.154^ × age^−0.203^ × 1.212 [if black] × 0.742 [if female] (17). EGFR < 60 ml/min/1.73 m^2^ was defined as chronic kidney disease (CKD), indicated by the Modification of Diet in Renal Disease (MDRD) Study (18). Marital status included living alone (unmarried, separated, divorced, and widowed) and not living alone (living with a partner and married). All details of study variables and covariates in the present study could be accessed through the webpage www.cdc.gov/nchs/nhanes.

### Statistical Analysis

Statistical analysis was performed using R version 4.1.0. Considering the complex design of study of NHANES, we applied the survey package in R to calculate sample-weighted prevalence and other statistics to study the population characteristics of American wrist fracture patients. In addition, generalized linear model was used to analyze variables including age, gender, race, education, marital status, smoking, drinking, hypertension, diabetes mellitus, heart failure, obesity, chronic kidney diseases, osteoporosis, medication history of prednisone or cortisone, history of parental wrist fracture, and univariate and multivariate relationships with wrist fractures. The covariate with *p*-value < 0.20 in univariate analysis was included in the multivariate logistic regression analysis to study risk factors related to wrist fracture. *p* < 0.05 (two-sided) was considered statistically significant.

## Results

### Clinical Characteristics

Clinical characteristics of the study population are displayed in **Table 1**. Men took up 46.5% of the total cases while women took up 53.5%. The weighted mean age was 63.7. Other Hispanics, non-Hispanic blacks, and other races including multi-racial, as well as never smokers and participants with a history of parental wrist fracture were higher in the wrist fracture group; serum calcium and serum phosphorus levels were lower in the wrist fracture group compared to the non-wrist fracture group. Non-Hispanic whites and participants with osteoporosis, obesity, daily oral administration of cortisone or prednisone, frequent drinking, and current smoking were more likely to be present in the non-wrist fracture group.

**Table 1:** Clinical characteristics of the study population.

Parameters	Total mean or % [95% CI]	Non-fracture mean or % [95% CI]	Fracture mean or % [95% CI]	*p*-value
N*	5,842	5,288	554	–
Age (mean, years)	63.7 [63.2,64.2]	63.7 [63.2,64.2]	63.8 [62.7,64.9]	0.8709
Sex (%)				
Male	46.5 [45.1,48]	46.1 [44.7,47.5]	46.1 [44.7,47.5]	0.2676
Female	53.5 [52,54.9]]	53.9 [52.5,55.3]	50.7 [45,56.3]	0.2676
Race (%)				
Mexican American	5.4 [3.9,7.4]	5.7 [4.1,7.8]	3.4 [1.8,6.3]	0.0699
Other Hispanic	4.7 [3.8,5.8]	4.9 [4.0,6.1	2.8 [2.0,4.0]	0.0014
Non-Hispanic White	72 [67.9,75.8]	70.4 [66,74.5]	83.6 [79.4,87]	<0.0001
Non-Hispanic Black	10.2 [8.2,12.5]	10.7 [8.6,13.3]	5.9 [4.5,7.7	0.0001
Other Race—Including Multi-Racial	7.7 [6.3,9.5]	10.7 [8.6,13.3]	5.9 [4.5,7.7]	0.0042
Marital status (%)				
Living alone	33.9 [31.9,35.9]	35.3 [32.9,37.7]	35 [30.7,39.5]	0.9011
Not living alone	36.7 [34.9,38.5]	64.7 [62.3,67.1]	65 [60.5,69.3]	0.9011
Level of education (%)				
Less than high school	13.9 [17.8,16.2]	14.2 [12,16.8]	11.4 [8.4,15.3]	0.1985
High school or than	86.1 [80.7,91.6]	85.8 [79.7,91.9]	88.6 [78.0,99.5]	0.1225
Drinking (%)				
Never	32.3 [29.7,35]	32.7 [30.2,35.3]	29.5 [23.6,36.1]	0.2763
Occasional drink	55.6 [52.6,58.5]	56.3 [53.4,59.1]	50.5 [43.9,57.1]	0.0607
Frequent drink	12.1 [10.7,13.7]	11 [9.8,12.4]	20 [15,26.3]	0.0003
Smoking status (%)				
Never smoker	53.5 [50.7,56.3]	54.9 [52.3,57.5]	43.3 [35.7,51.1]	0.003
Past smoker	32.2 [30.1,34.3]	31.6 [29.5,33.8]	36.2 [29.5,43.5]	0.1823
Current smoker	14.3 [12.6,16.3]	13.5 [11.8,15.3]	20.5 [15.9,26.1]	0.0016
Obesity (%)	42 [39.6,44.4]	41.3 [39,43.6]	46.8 [40.8,52.9]	0.049
Chronic kidney disease (%)	50.5 [48.9,52.2]	50.5 [48.9,52.2]	50.6 [44.9,56.2]	0.989
Hypertension (%)	72.6 [70.6,74.6	72.6 [70.3,74.7]	72.9 [66.7,78.3]	0.9277
Diabetes mellitus(%)	22 [20.8,23.3]	21.7 [20.4,23.1]	23.9 [19.8,28.5]	0.334
Heart failure (%)	4.6 [3.9,5.5]	4.5 [3.8,5.4]	5.6 [3.6,8.4]	0.3034
Stroke (%)	5.9 [5.2,6.8]	5.8 [5,6.7]	6.7 [4.5,10]	0.5205
Every taken prednisone or cortisone daily (%)	7.5 [6.5,8.5]	6.9 [5.9,8.1]	11.3 [7.7,16.4]	0.0345
Osteoporosis (%)	10.4 [9.1,11.8]	9.3 [8.2,10.5]	18.4 [13.6,24.5]	<0.0001
History of parental wrist fractures (%)	17.2 [15.7,18.7]	83.5 [81.7,85.2]	77.6 [73.3,81.4]	0.0123
Serum phosphate (mg/dl)	3.18 [3.12, 3.31]	3.45 [3.23, 3.48]	3.08 [2.94, 3.24]	0.046
Serum calcium (mg/dl)	9.3 [8.2,11.4]	10.2 [8.6,12.5]	9.0 [8.45,10.1]	0.0746
Body mass index (kg/m^2^)	28.2 [28.0,28.4]	27.6 [27.1,28.2]	29.2 [29.0,29.4]	0.078

*Unweighted calculation.

### Population Characteristics of Wrist Fractures From NHANES, 2013–2014, and 2017–2018

**Figure 1:**
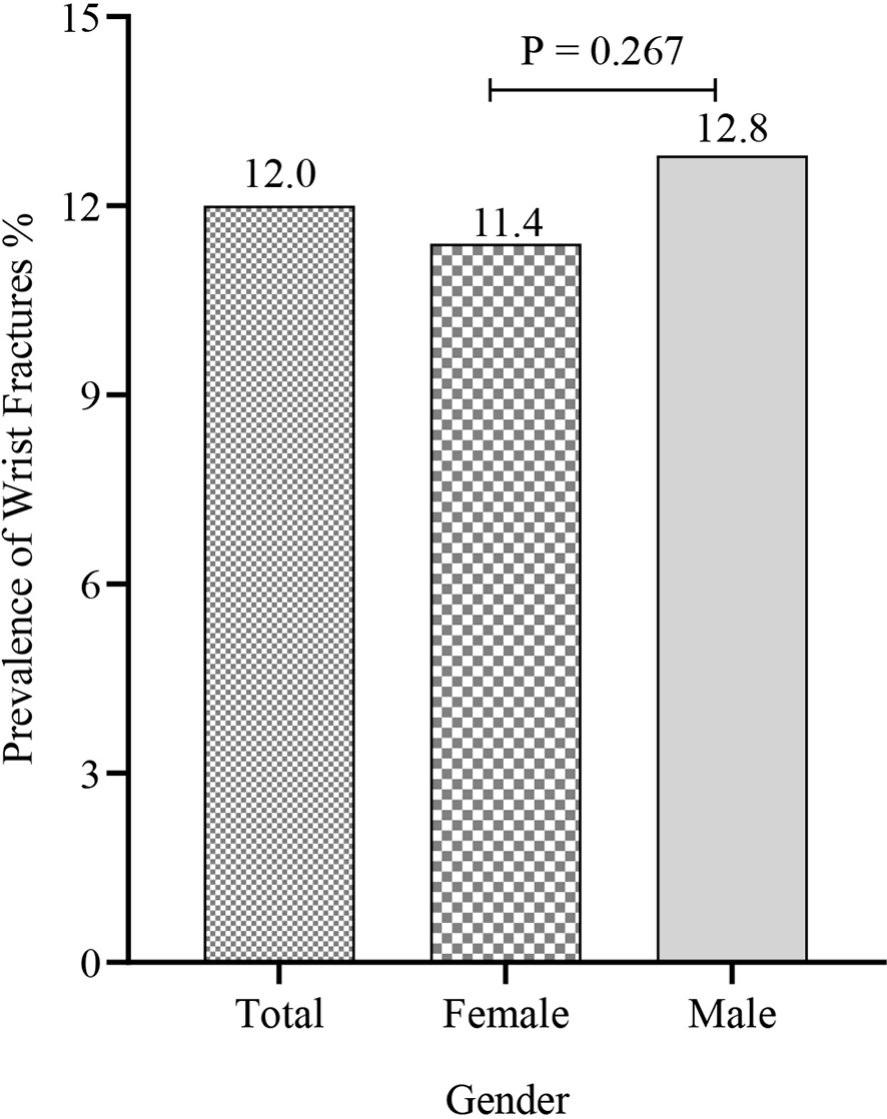
Relationship between gender and prevalence of wrist fractures.

**Figure 2:**
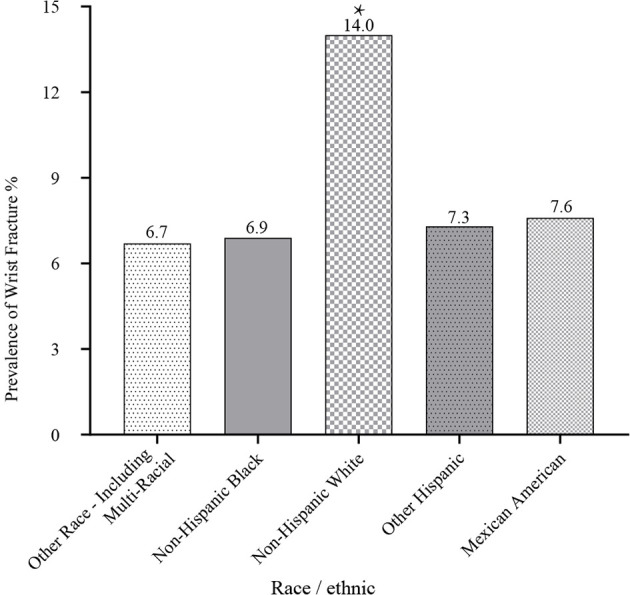
Relationship between race/ethnic and prevalence of wrist fractures. *p < 0.05 non-Hispanic white vs. other race/ethnic (Mexican Americans, other Hispanics, non-Hispanic blacks, and other races including multi-racial).

**Figure 3:**
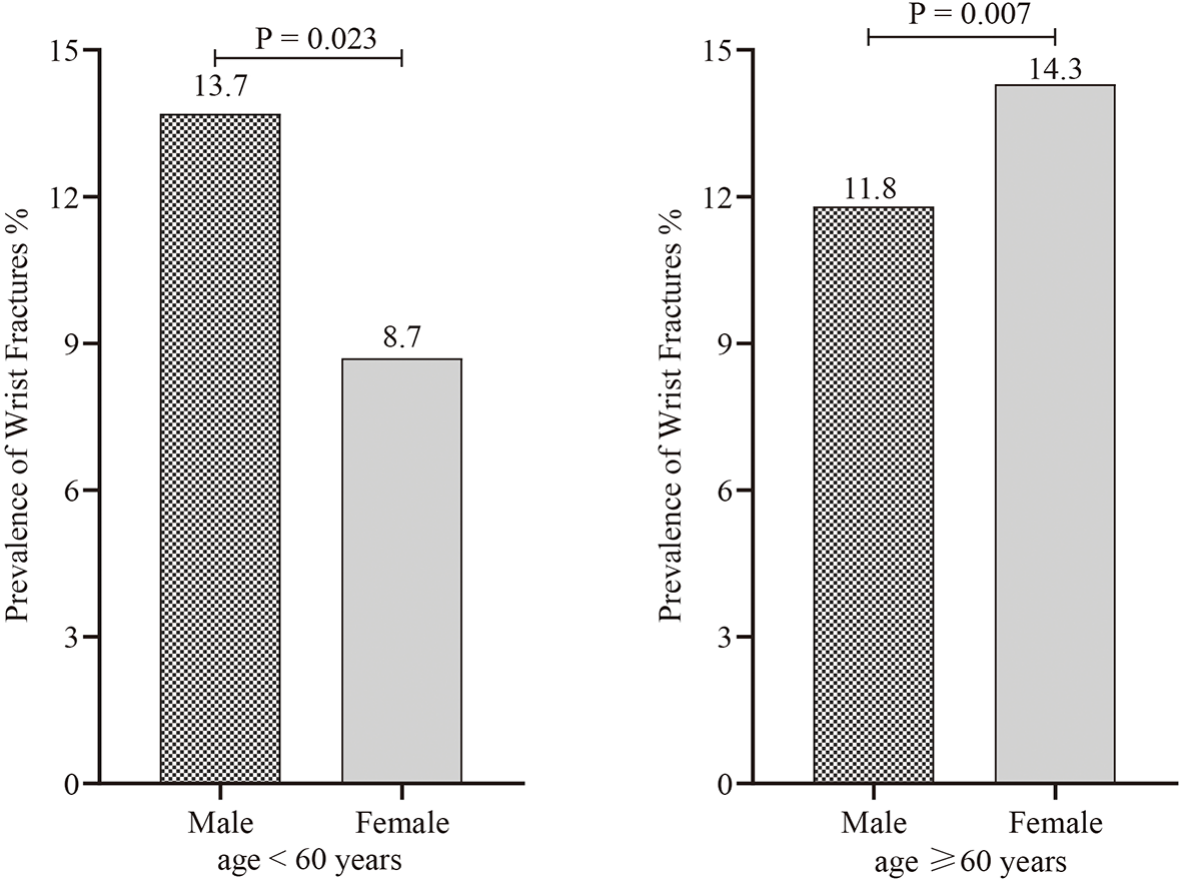
The difference in the prevalence of wrist fractures between the sexes when the first wrist fracture occurred at age < 60 years and age ≥ 60 years.

Among the total 5,842 US participants ≥ 50 years of age, 554 patients with wrist fractures were identified *via* standard questionnaire described above. The prevalence of wrist fractures was 12%, with similarities in men and women (men 12.8% vs. women 11.4%, *p* = 0.267) (**Figure 1** and **Additional file: Table S1**). Non-Hispanic whites had the highest wrist fracture prevalence of 14% and there was a significant difference in prevalence between non-Hispanic whites and other ethnic groups (Mexican Americans: 7.6%, Other Hispanics: 7.3%, Non-Hispanic blacks: 6.9%, Other Race—Including Multi-Racial: 6.7%) (**Figure 2** and **Additional file: Tables S2, S3**). Among the wrist fracture cases, a fall from a standing height or less (56%) or a hard fall (34.8%) was the leading cause of the first wrist fracture; 17.8% of the population experienced a second wrist fracture and 1.7% experienced four or more fractures. However, there was no gender difference in the cause and frequency (**Table 2**). **Figure 3** demonstrates the difference in the prevalence of wrist fractures between the sexes when the first wrist fracture occurred before age 60 and after age 60. The prevalence of wrist fracture was higher in men than in women before the age of 60 years when the first wrist fracture occurred (13.7% vs. 8.7%, *p* = 0.023). However, after the age ≥ 60 years, the prevalence in women was higher than in men (11.8% vs. 14.3%, *p* = 0.007).

**Table 2:** Epidemiological characteristics of wrist fractures in the population aged 50 years or older.

Parameters	TotalMean or % [95% CI]	MaleMean or % [95% CI]	FemaleMean or % [95% CI]	*p*-value
*N* ^*^	554	239	315	0.029
Age when 1st time wrist fracture occurred (years)	29.4 [27.7,31.1]	24.3 [22.1,26.5]	34.5 [31.5,37.6]	<0.0001
Factors of 1st time wrist fracture (%)				0.1028
A fall from a standing height or less^#^	56 [46.2,65.4]	42.5 [24.4,63]	60 [47.9,71.1]	
A hard fall^§^	34.8 [26.7,43.8]	38.1 [24.7,53.5]	33.8 [23,46.7]	
A car accident or other severe trauma	8.5 [4.6,15.3]	17.6 [7.2,36.9]	5.8 [2.9,11.2]	
Don’t know	0.7 [0.2,2.8]	1.8 [0.2,13.2]	0.3 [0,2.5]	
Frequency of wrist fractures (%)				0.3687
Twice	17.8 [11.2,27.3]	15.7 [8.6,32.6]	19.9 [12.1,33.1]	
Three times	5.5 [3.4.11.2]	5.6 [1.9,17.8]	5.4 [2.2,12.4]	
Four times or more	2.2 [0.9,5.2]	2.5 [0.6,10.3]	1.9 [0.8,4.1]	

^*^Unweighted calculation.

^#^A fall from a standing height or less, for example, tripped, slipped, fell out of bed.

^§^A hard fall such as falling off a ladder or step stool, or down the stairs.

### The Relationship Between Each Variable and Wrist Fracture

Univariate analysis of the wrist fractures and demographic characteristics of American men and women aged ≥ 50 are shown (**Table 3**): correlated risk factors included frequent drinking, smoking, daily oral administration of cortisone, concomitant osteoporosis, and fractures in family history and obesity.

**Table 3:** Univariate and multivariate analysis of wrist fracture.

Parameters	Univariate analysis		Multivariate analysis	
	OR [95% CI]	*p*-value	OR [95% CI]	*p*-value
Age	1.00 [0.99,1.01]	0.8709	–	
Sex				
Male	1 [ref]		–	
Female	0.88 [0.70,1.10]	0.2676	–	
Race				
Mexican American	1 [ref]		1 [ref]	
Other Hispanic	0.95 [0.62,1.457]	0.8196	0.97 [0.66,1.41]	0.8558
Non-Hispanic White	1.96 [1.12,3.43]	0.0255	1.78 [1.04,3.04]	0.0534
Non-Hispanic Black	0.90 [0.54,1.52]	0.7018	0.80 [0.47,1.37]	0.4350
Other Race—Including Multi-Racial	0.87 [0.41,1.85]	0.7236	0.82 [0.39,1.72]	0.6041
Marital status				
Living alone	1 [ref]		–	
Not living	0.99 [0.82,1.19]	0.9011	–	
Level of education				
Less than high school	1 [ref]		–	
High school or than	1.22 [0.81, 1.73]	0.31	–	
Drinking (%)				
Never	1 [ref]		1 [ref]	
Occasional drink	0.99 [0.76,1.31]	0.9734	1.35 [0.89, 2.03]	0.1734
Frequent drink	2.02 [1.36,2.99]	0.0015	1.96 [1.36, 2.81]	0.0027
Smoking status (%)				
Never smoker	1 [ref]		1 [ref]	
Past smoker	1.45 [1.05,2.03]	0.0347	0.89 [0.65, 1.22]	0.4751
Current smoker	1.93 [1.41,2.65]	0.0003	1.81 [1.32, 2.65]	0.0856
Hypertension				
No	1 [ref]		–	
Yes	1.01 [0.74,1.39]	0.9277	–	
Diabetes mellitus				
NO	1 [ref]		–	
YES	1.13 [0.88,1.45]	0.3339	–	
Heart failure				
No	1 [ref]		–	
Yes	1.25 [0.82,1.91]	0.3034	–	
Stroke				
No	1 [ref]		–	
Yes	1.16 [0.74,1.84]	0.5205	–	
Ever taken prednisone or cortisone daily				
No	1 [ref]		1 [ref]	
Yes	1.71 [1.06,2.75]	0.0345	1.45 [0.91,2.30]	0.1458
Osteoporosis				
No	1 [ref]		1 [ref]	
Yes	2.21 [1.61,3.05]	0	2.40 [1.72, 3.37]	0.0002
History of parental wrist fractures				
NO	1 [ref]		1 [ref]	
YES	1.46 [1.11,1.94]	0.0123	1.20 [0.85, 1.685]	0.3240
Obesity				
No	1 [ref]		1 [ref]	
Yes	1.25 [1.01,1.55]	0.049	1.22 [1.06, 1.59]	0.0278
Chronic kidney diseases				
No	1 [ref]		–	
Yes	1.00 [0.80,1.25]	0.989	–	
Serum phosphate	1.20 [1.02, 1.43]	0.003	1.25 [1.11, 1.43]	0.0340
Serum calcium	0.91 [0.81, 1.20]	0.457	–	

Multivariate analysis of the wrist fractures and demographic characteristics of American men and women aged ≥ 50 are shown (**Table 3**): frequent drinking (OR: 1.96, 95% CI: 1.36–2.81), current smoker (OR: 1.81, 95% CI: 1.32–2.65), serum phosphate level (OR: 1.25, 95% CI: 1.11–1.43), osteoporosis (OR: 2.40, 95% CI: 1.72–3.37), and obesity (OR: 1.22, 95% CI: 1.06–1.59) were independent risk factors associated with wrist fractures (**Table 3**).

### Subgroups

The analysis of sex and race subgroups was conducted. Non-Hispanic white women were positively associated with the occurrence of wrist fractures (**Additional File: Table S4**). Osteoporosis, frequent drinking, and high serum phosphate level were risk factors of wrist fractures in all races. Current smokers among Mexican Americans, non-Hispanic whites, and other race including multi-racial were significantly more likely to experience wrist fractures. Mexican Americans, other Hispanics, and non-Hispanic whites with obesity were more susceptible to wrist fractures (**Additional file: Table S5**).

## Discussion

In the present cross-sectional study of 5,842 Americans ≥ 50 years of age, the prevalence of wrist fractures was 12%, without gender difference. Non-Hispanic whites had a higher risk of wrist fractures than other races. In addition, this study also showed that the first wrist fracture was mostly caused by a fall from a standing height (56%) or a hard fall (34.8%). Among those who had experienced their first wrist fracture, 17.8% had experienced a second wrist fracture, while 1.7% had experienced four or more fractures. The difference in the prevalence of wrist fractures between male and female was related to the age at which the first fracture occurred. Before the age of 60, men had a higher prevalence of their first wrist fracture than women. However, the situation became the opposite after the age of 60. Obesity, frequent drinking, current smoking, high serum phosphate level, non-Hispanic white women, and osteoporosis in women in the wrist fracture population were positively associated with wrist fractures. To the best of our knowledge, our study conducted the first research on patients with wrist fractures above the age of 50 based on data extracted from the NHANES, which enlightens the understanding of the disease as well as lends insight into prevention and improving the prognosis.

In 2000, WHO reported that the prevalence of wrist fracture in the total population whose ages ≥ 50 was 18.5% (19). Hye-Young Kwon et al. suggested that the prevalence in Korea was 4.52% with no gender difference (20). In a retrospective study of pediatric anterior wall fractures, the prevalence of forearm fractures in children aged 0 to 17 years was 24.1% in Washington, D.C (21). In the Oslo Health Study conducted on 5,976 participants, the prevalence of anterior wall fractures was 7.2% in men and 10.1% in women between the age of 59 and 60, and was 9.1% in men and 34.5% in women between the age of 75 and 76 (22). The Study of Women’s Health Across the Nation suggested that the prevalence of forearm fractures in postmenopausal women was 15% (23). The reason why these results differed from our result (prevalence of 12%) might be due to the different parameters included in the studied cases, such as age, region, and the initial occurrence time. Furthermore, we discovered that non-Hispanic whites had a higher risk of fracture, which was consistent with the results of a study of fracture risk in the diabetic population (24). The higher risk that non-Hispanic whites experienced might be related to race-specific genes and dietary characteristics (25). We also found that the first fracture presented earlier in women than in men, which was supported by the Oslo Health study (22). This phenomenon may be attributed to the menopausal period in women age above 50 (6, 13, 26–28).

In addition, this study also showed that a fall from a standing height was a major contributing factor to forearm fractures (56.0%), which was in line with the results reported by Norma J Mac Intyre and K C Chung (29, 30). People in their adolescence (under the age of 25) are more exposed to various activities and movements that have a potential danger of causing physical injuries, which included falling from a standing height (31–33). Therefore, active anti-osteoporosis treatment and various effective measures for preventing falls, such as wearing non-slip shoes, avoiding slippery road surface, and strengthening physical exercise, are vital for preventing forearm fractures. This study also discovered that sex differences in the prevalence of wrist fractures were associated with age at the time of fracture. The prevalence of wrist fractures was higher in men than in women when the first wrist fracture occurred before the age of 60, which was supported by previous epidemiological studies of wrist fractures in which young men were more likely to have wrist fractures than young women (34, 35). However, as women age, especially after menopause, they tend to lose more bone mass than men, which makes them more likely to suffer from wrist fractures (36, 37).

Obesity, smoking, drinking alcohol, high serum phosphate level, usage of corticosteroids, and osteoporosis are common factors associated with fractures (4, 38, 39). In our study, we have verified that obesity, occasional smoking, frequent smoking, excessive drinking, osteoporosis, and prednisolone intake accounted for a higher proportion in the population with wrist fractures. Smoking was an important risk factor for non-vertebral fracture in diabetic women, and the risk of non-vertebral fracture in diabetic women who have smoked is 3.47 times higher than that in non-smoking diabetic women (40). Smoking can promote fractures by reducing bone mineral density in a dose-dependent manner (41). Our study revealed that drinking alcohol also caused wrist fractures by affecting bone mineral density. Drinking alcohol was independently associated with an increased risk of forearm fractures in a dose–response manner. Compared with non-drinkers, the RR for forearm fractures was 1.38 for women who drank greater than or equal to 25 g daily (42). We further revealed the importance of smoking cessation and alcohol restriction for the prevention of hip fracture in the elderly. The positive association between osteoporosis and wrist fracture was only in women, presumably because as women age, estrogen decreases and BMD loss is more severe than in men, increasing the risk of fracture (43, 44). In line with the Osteoporotic Fractures in Men (MrOS) study, our study suggested that increased serum phosphate level can also increase the risk of wrist fractures (13). Increased serum phosphate level leads to decreased calcium absorption and may reflect hypoparathyroidism and liver disease, all of which are associated with osteoporosis, bone loss, and fracture risk (13, 45). Another interesting finding was that female non-Hispanic whites had an elevated risk of fractures (46). The mechanism for this may be gender and racial differences in serum 25(OH)D and BMD. Results of the 2003–2004 National Health and Nutrition Examination Survey (NHANES) showed that when serum 25(OH)D decreased in whites, BMD decreased significantly as well. However, such change was not observed in blacks (47).

The study conducted by Jason Lacombe et al. showed that high BMI was associated with a reduced risk of forearm and wrist neck fractures compared to women with an ideal BMI of 20.0 to 24.9 kg/m^2^ (48). The fact that a high BMI is protective and that weight loss appears to increase the risk of fractures may indicate an interaction between bone and fat (49).

Some limitations in this study should be addressed. First, this study could not avoid reporting bias and memory bias, such as the history of wrist fractures, medication history of prednisone or cortisone, and smoking and drinking history. Second, due to the design of a cross-sectional study, relative conclusions of causality could not be obtained. Finally, some variables that were closely related to fracture, such as activity level, use of vitamin D and/or calcium in dietary supplements, and BMD, were not considered as covariates because they involved too many missing values. Despite the discussed limitations, the overall data collected from the NHANES database were reliable and had undergone sufficient validation to filter accurate individual samples to determine the prevalence.

## Conclusions

In conclusion, our study demonstrated that the prevalence of wrist fractures in Americans aged 50 and above was 12%. The prevalence was similar between male and female patients. Moreover, falling from a standing height was the main cause of the first wrist fracture. Frequent drinking, current smoker, high serum phosphate level, osteoporosis, and obesity were the risk factors for wrist fractures. In women, non-Hispanic whites are more likely to experience wrist fractures. Stratified by race, smoking was negatively associated with wrist fracture in other Hispanics and non-Hispanic blacks, while obesity was a risk factor of wrist fractures in Mexican Americans, other Hispanics, and non-Hispanic whites.

## Data Availability Statement

The raw data supporting the conclusions of this article will be made available by the authors, without undue reservation.

## Author Contributions

This study was designed by JN. JY extracted the associated data from NHANES. QL performed statistical analysis. JY and QL completed the composition of the manuscript, helped supervised the analysis, and revised and approved the manuscript. All authors contributed to the article and approved the submitted version.

## Conflict of Interest

The authors declare that the research was conducted in the absence of any commercial or financial relationships that could be construed as a potential conflict of interest.

## Publisher’s Note

All claims expressed in this article are solely those of the authors and do not necessarily represent those of their affiliated organizations, or those of the publisher, the editors and the reviewers. Any product that may be evaluated in this article, or claim that may be made by its manufacturer, is not guaranteed or endorsed by the publisher.

## Abbreviations

NHANES, National Health and Nutrition Examination Survey; CI, Confidence interval; OR, Odds ratio; BMI, Body mass index; EGFR, Estimate the glomerular filtration rate; CKD, Chronic kidney disease.
