# Supplementary material for: Prevalence, Characteristics, and Associated Risk Factors of Wrist Fractures in Americans Above 50: The Cross-Sectional NHANES Study
Source: Front Endocrinol (Lausanne). 2022 Apr 25;13:800129. doi: 10.3389/fendo.2022.800129 (PMC9082306; doi:10.3389/fendo.2022.800129)
Supplement: Supplementary file 1 [file DataSheet_1.pdf]

Prevalence, characteristics, and associated risk factors of Wrist fractures in Americans above 50: the cross-sectional NHANES study

Juncai Ye [1], Qiao Li [2] and Jing Nie [3*]

[1]Orthopedics Department, Zhejiang Hospital, Hangzhou, Zhejiang, China

[2]The Second Clinical Medical College of Zhejiang Chinese Medical University, Hangzhou, Zhejiang Province, China

[3]Department of Orthopedics, Traumatology and Orthopedics Hospital of Traditional Chinese Medicine of Xiaoshan District, Hangzhou, Zhejiang Province, China

Correspondence[*]: Jing Nie, Orthopedics Department, Traumatology and Orthopedics Hospital of Traditional Chinese Medicine of Xiaoshan District，Hangzhou, Zhejiang Province 311261, China.

E-mail: drjingnie@126.com

Supplement

Table S1 Relationship between gender and prevalence of wrist fractures

Sex	Total	Male	Female	P value
Wrist Fracture % [95% CI]	12.0[10.7,13.5]	12.8[10.8,15.0]	11.4[9.9,13.2]	0.267

Table S2 Relationship between race and prevalence of wrist fractures.

Race	Mexican-American	Other Hispanic	Non-Hispanic White	Non-Hispanic Black	Other Race- Including Multi-Racial
Wrist Fracture % [95% CI]	7.6[4.5,12.7]	7.3[5.7,9.3]	14.0[12.2,16.0]	6.9[5.4,8.8]	6.7[4.3,10.3]

Table S3 Comparisons of prevalence of wrist fracture among race

Parameters	P value
Other Hispanic vs Mexican American	0.966742
Non-Hispanic White vs Mexican American	0.000065
Non-Hispanic Black vs Mexican American	0.99975
Other Race - Including Multi-Racial vs Mexican American	0.852419
Non-Hispanic White vs Other Hispanic	0.008834
Non-Hispanic Black vs Other Hispanic	0.896913
Other Race - Including Multi-Racial vs Other Hispanic	0.486371
Non-Hispanic Black vs Non-Hispanic White	<0.000001
Other Race - Including Multi-Racial vs Non-Hispanic White	<0.000001
Other Race - Including Multi-Racial vs Non-Hispanic Black	0.863373

Table S4 univariate and multivariate analysis of Wrist Fracture in sex

Parameters	Univariate analysis		Multivariate analysis	
	OR[95CI]	P value	OR[95CI]	P value
Male				
Age	0.99[0.98, 1.01]	0.4868	-	
Race				
Mexican American	1[reference]		1[reference]	
Other Hispanic	0.7[0.3, 1.6]	0.4008	0.73[0.32, 1.66]	0.4582
Non-Hispanic White	1.77[0.9, 3.5]	0.1108	1.85[0.92, 3.69]	0.0974
Non-Hispanic Black	0.92[0.5, 1.69]	0.787	0.82[0.4, 1.65]	0.5798
Other Race - Including Multi-Racial	0.78[0.28, 2.14]	0.6279	0.75[0.26, 2.14]	0.5997
Marital status				
Living alone	1[reference]		-	
Not living	0.79[0.52, 1.18]	0.2564	-	
Level of education				
Less than high school	1[reference]		-	
High school or than	1.15[0.62, 2.12]	0.6593	-	
Drinking				
Never	1[reference]		1[reference]	
Occasional drink	1.01[0.61, 1.68]	0.9591	1.08[0.74, 1.45]	0.8647
Frequent drink	1.32[1.05, 2.19]	0.0412	1.47[1.12, 2.25]	0.0412
Smoking status				
Never smoker	1[reference]		1[reference]	
Past smoker	1.54[0.96, 2.49]	0.0845	1.52[0.94, 2.45]	0.1036
Current smoker	1.47[0.89, 2.43]	0.1449	1.74[1.07, 2.81]	0.0351
Hypertension				
NO	1[reference]		-	
YES	0.85[0.56, 1.28]	0.4448	-	

Diabetes mellitus				
NO	1[reference]		1[reference]	
YES	1.34[1.01, 1.78]	0.0515	1.33[0.99, 1.79]	0.0705
Heart failure				
NO	1[reference]		-	
YES	1.15[0.69, 1.92]	0.6004	-	
Stroke				
NO	1[reference]		-	
YES	1.02[0.55, 1.9]	0.9521	-	
Every taken prednisone or cortisone daily				
NO	1[reference]		1[reference]	
YES	1.6[0.86, 2.97]	0.1459	1.48[0.73, 2.99]	0.2843
Osteoporosis				
NO	1[reference]		1[reference]	
YES	1.37[1.12, 2.92]	0.0163	1.24[1.05, 2.45]	0.0463
History of Parents wrist fractures				
NO	1[reference]		-	
YES	1.41[0.76, 2.61]	0.2794	-	
Obesity				
NO			1[reference]	
YES	1.45[1.03, 2.02]	0.0403	1.32[1.14, 1.86]	0.0077
Chronic kidney diseases				
NO	1[reference]		-	
YES	1.11[0.54, 2.3]	0.7807	-	
Serum phosphate	1.18[1.01, 1.35]	0.014	1.56[1.24, 1.78]	0.0247
Serum calcium	0.94[0.87, 1.22]	0.267	-	
Female				
Age	1.01[1.0, 1.03]	0.0892	1.01[0.99, 1.03]	0.2237
Race				
Mexican American	1[reference]		1[reference]	
Other Hispanic	1.29[0.77, 2.17]	0.3497	1.29[0.78, 2.14]	0.3329
Non-Hispanic White	2.26[1.29, 3.98]	0.0088	2.08[1.13, 3.84]	0.0314
Non-Hispanic Black	0.93[0.48, 1.82]	0.8428	0.94[0.71, 1.78]	0.6452
Other Race - Including Multi-Racial	1.01[0.49, 2.11]	0.9742	1.09[0.54, 2.17]	0.8175
Marital status				
Living alone	1[reference]		-	
Not living	1.21[0.91, 1.62]	0.2001	-	
Level of education				
Less than high school	1[reference]		-	
High school or than	1.15[0.64, 2.06]	0.6428	-	
Drinking				

Never	1[reference]		1[reference]	
Occasional drink	0.94[0.74, 1.19]	0.6248	0.88[0.65, 1.2]	0.4389
Frequent drink	4.16[2.53, 6.85]	< 0.0001	3.39[1.75, 6.53]	0.002
Smoking status				
Never smoker	1[reference]		1[reference]	
Past smoker	1.25[0.79, 1.98]	0.3394	1[0.59, 1.68]	0.9991
Current smoker	2.41[1.59, 3.65]	0.0003	1.72[1.06, 2.8]	0.0423
Hypertension				
NO	1[reference]		-	
YES	1.21[0.72, 2.03]	0.4719	-	
Diabetes mellitus				
NO	1[reference]		-	
YES	0.91[0.62, 1.34]	0.6386	-	
Heart failure				
NO	1[reference]		-	
YES	1.36[0.7, 2.64]	0.3735	-	
Stroke				
NO	1[reference]		-	
YES	1.3[0.8, 2.12]	0.3014	-	
Every taken prednisone or cortisone daily				
NO	1[reference]		1[reference]	
YES	1.83[0.97, 3.47]	0.0736	1.64[0.9, 3.01]	0.1253
Osteoporosis				
NO	1[reference]		1[reference]	
YES	2.78[2, 3.88]	<0.0001	2.32[1.69, 3.17]	<0.0001
History of Parents wrist fractures				
NO	1[reference]		1[reference]	
YES	1.59[1.12, 2.24]	0.0138	1.24[0.8, 1.92]	0.347
Obesity				
NO	1[reference]		1[reference]-	
YES	1.1[1.02, 1.45]	0.0406	1.08[1.03, 1.26]	0.0325
Chronic kidney diseases				
NO	1[reference]		1[reference]	
YES	1.01[0.98, 1.31]	0.0834	1.12[0.94, 1.56]	0.065
Serum phosphate	1.27[1.08, 1.51]	0.038	1.42[1.31, 1.71]	0.0046
Serum calcium	0.94[0.87, 1.22]	0.267	-	

Table S5 univariate and multivariate analysis of Wrist Fracture in race

Parameters	Univariate analysis		Multivariate analysis	
	OR[95CI]	P value	OR[95CI]	P value
Mexican American				
Age	1.01[0.97, 1.04]	0.6409	-	
Sex				
men	1[reference]		-	
Female	0.75[0.42, 1.35]	0.3422	-	
Marital status				
Living alone				
Not living	1.02[0.56, 1.86]	0.9585		
Level of education				
Less than high school	1[reference]		-	
More than high school	0.47[0.14, 1.61]	0.2298	-	
Drinking				
Never	1[reference]		1[reference]	
Occasional drink	1.33[0.72, 2.47]	0.3666	1.21[0.84, 2.12]	0.1576
Frequent drink	1.83[1.3, 2.3]	0.0229	1.42[1.21, 1.8]	0.0345
Smoking status				
Never smoker	1[reference]		1[reference]	
Past smoker	1.01[0.38, 2.74]	0.9783	1.14[0.92, 1.57]	
Current smoker	1.46[0.88, 2.22]	0.1329	1.55[1.27, 2.1]	0.0025
Hypertension				
NO	1[reference]		-	
YES	1.14[0.58, 2.23]	0.7077	-	
Diabetes mellitus				
NO	1[reference]		-	
YES	0.7[0.37, 1.34]	0.2847	-	
Heart failure				
NO	1[reference]		1[reference]	
YES	2.48[0.82, 7.52]	0.1077	1.49[0.4, 5.51]	0.5481
Stroke				
NO	1[reference]		-	
YES	1.63[0.47, 5.63]	0.4371	-	
Every taken prednisone or cortisone daily				
NO	1[reference]		-	
YES	0.88[0.2, 3.79]	0.861	-	
Osteoporosis				
NO	1[reference]		1[reference]	
YES	2.59[1.14, 5.85]	0.0227	3.81[1.44, 10.04]	0.0068
History of Parents wrist				

fractures				
NO	1[reference]		-	
YES	1.25[0.54, 2.87]	0.6068	-	
Obesity				
NO	1[reference]		1[reference]	
YES	2.01[1.07, 3.74]	0.0287	2.08[1.1, 3.95]	0.0248
Chronic kidney diseases				
NO	1[reference]		1[reference]	
YES	0.67[0.36, 1.22]	0.1921	1.37[0.88, 1.75]	0.1271
Phosphate	2.83[1.62, 4.92]	0.0002	1.78[1.24, 3.63]	0.004
Calcium	1.04[0.67, 1.6]	0.8714	-	
Other Hispanic				
Age	1.01[0.99, 1.03]	0.3422	-	
Sex				
men	1[reference]		-	
Female	2191323.45[0, Inf]	0.9841	-	
Marital status				
Living alone	1[reference]		-	
Not living	0.79[0.38, 1.64]	0.5353	-	
Level of education				
Less than high school	1[reference]		-	
More than high school	0.63[0.23, 1.72]	0.3705	-	
Drinking				
Never	1[reference]		1[reference]	
Occasional drink	1.1[0.52, 2.34]	0.8018	0.87[0.44, 1.71]	0.6898
Frequent drink	1.01[0.89, 1.5]	0.1292	1.11[1.02, 2.6]	0.0467
Smoking status				
Never smoker	1[reference]		-	
Past smoker	0.92[0.41, 2.05]	0.8417	-	
Current smoker	1.49[0.5, 4.42]	0.4744	-	
Hypertension				
NO	1[reference]		-	
YES	2.09[0.34, 12.74]	0.4238	-	
Diabetes mellitus				
NO	1[reference]		-	
YES	0.97[0.46, 2.03]	0.9295	-	
Heart failure				
NO	1[reference]		-	
YES	1.56[0.67, 3.64]	0.2989	-	
Stroke				
NO	1[reference]		-	
YES	0.86[0.4, 1.87]	0.7081	-	
Every taken prednisone or cortisone daily				

NO	1[reference]		-	
YES	1.89[0.56, 6.47]	0.3074	-	
Osteoporosis				
NO	1[reference]		1[reference]	
YES	2.84[1.06, 7.61]	0.0375	1.06[1.01, 1.24]	0.04
History of Parents wrist fractures				
NO	1[reference]		-	
YES	0.94[0.35, 2.53]	0.9045	-	
Obesity				
NO	1[reference]		1[reference]	
YES	1.87[0.94, 3.73]	0.0736	1.01[3.65, 0.05]	0.05
Chronic kidney diseases				
NO	1[reference]		-	
YES	0.94[0.91, 1.02]	0.984	1[reference]	
Phosphate	1.91[1.42, 2.59]	<0.001	1.91[1.42, 2.59]	
Calcium	1.1[0.88, 1.38]	0.3857	-	
Other Hispanic				
Age	1.48[0.98, 2.23]	0.0615	0.86[0.42, 1.77]	0.6813
Sex				
men	1[reference]		1[reference]	
Female	0.6[0.32, 1.11]	0.1029	1.89[1.33, 3.05]	0.0425
Marital status				
Living alone			-	
Not living	0.97[0.74, 1.26]	0.8099	-	
Level of education				
Less than high school	1[reference]		-	
More than high school	1.11[0.71, 1.74]	0.6436	-	
Drinking				
Never	1[reference]		1[reference]	
Occasional drink	0.88[0.65, 1.18]	0.3865	0.96[0.73, 1.28]	0.7925
Frequent drink	1.82[1.24, 2.66]	0.0022	1.97[1.36, 2.85]	0.0003
Smoking status				
Never smoker	1[reference]		1[reference]	
Past smoker	1.2[0.89, 1.61]	0.2251	1.1[0.83, 1.47]	0.496
Current smoker	1.66[1.14, 2.4]	0.0074	1.53[1.08, 2.16]	0.0171
Hypertension				
NO	1[reference]		-	
YES	0.96[0.71, 1.29]	0.7746	-	
Diabetes mellitus				
NO	1[reference]		-	
YES	1.13[0.83, 1.54]	0.4458	-	
Heart failure				
NO	1[reference]		-	
YES	0.89[0.54, 1.48]	0.6625	-	
Stroke				
NO	1[reference]		-	

YES	0.83[0.51, 1.35]	0.4528	-	
Every taken prednisone or cortisone daily				
NO	1[reference]		-	
YES	1.66[1.11, 2.48]	0.0145	-	
Osteoporosis				
NO	1[reference]		1[reference]	
YES	2.04[1.42, 2.93]	0.0001	2.32[1.65, 3.26]	0
History of Parents wrist fractures				
NO	1[reference]		-	
YES	1.15[0.83, 1.59]	0.4091	-	
Obesity				
NO	1[reference]		1[reference]	
YES	1.34[1.03, 1.75]	0.0297	1.38[1.07, 1.77]	0.0122
Chronic kidney diseases				
NO	1[reference]		1[reference]	
YES	1.73[0.94, 3.17]	0.0762	1.68[0.93, 3.03]	0.0839
Phosphate	2.03[1.07, 3.85]	0.0297		
Calcium	1.16[0.73, 1.83]	0.5253	-	
Non-Hispanic Black				
Age	1[0.98, 1.02]	0.8769		
Sex				
men	1[reference]		1[reference]	
Female	0.67[0.39, 1.13]	0.1333	0.78[0.69, 1.52]	0.3165
Marital status				
Living alone	1[reference]		-	
Not living	1.19[0.74, 1.93]	0.4729	-	
Level of education				
Less than high school	1[reference]		-	
More than high school	1.26[0.67, 2.37]	0.4807	-	
Drinking				
Never	1[reference]		1[reference]	
Occasional drink	0.85[0.49, 1.48]	0.5737	0.87[0.69, 1.15]	0.1165
Frequent drink	1.65[0.82, 3.29]	0.1587	2.06[1.11, 3.81]	0.0212
Smoking status				
Never smoker	1[reference]		-	
Past smoker	1.29[0.72, 2.3]	0.3918	-	
Current smoker	1.33[0.71, 2.46]	0.3708	-	
Hypertension				
NO	1[reference]		-	
YES	0.75[0.26, 2.19]	0.6014	-	
Diabetes mellitus				
NO	1[reference]		-	

YES	1.14[0.68, 1.93]	0.6137	-	
Heart failure				
NO	1[reference]		-	
YES	0.91[0.46, 1.81]	0.7947	-	
Stroke				
NO	1[reference]		-	
YES	0.73[0.3, 1.76]	0.4844	-	
Every taken prednisone or cortisone daily				
NO	1[reference]		-	
YES	1.42[0.63, 3.16]	0.3956	-	
Osteoporosis				
NO	1[reference]		1[reference]	
YES	2.14[0.94, 4.89]	0.0713	2.46[1.22, 4.99]	0.0123
History of Parents wrist fractures				
NO	1[reference]		-	
YES	0.9[0.33, 2.49]	0.8457	-	
Obesity				
NO	1[reference]		1[reference]	
YES	1.08[0.98, 1.28]	0.1221	0.78[0.5, 1.23]	0.2934
Chronic kidney diseases				
NO	1[reference]		-	
YES	0.61[0.25, 1.47]	0.268	-	
Phosphate	2.42[1.35, 4.32]	0.0028		
Calcium	0.96[0.73, 1.27]	0.7969	-	
Other Race - Including Multi-Racial				
Age	0.64[0.2, 2.01]	0.4401	-	
Sex				
men	1[reference]		-	
Female	1.46[0.16, 13.49]	0.7393	-	
Marital status				
Living alone			-	
Not living	1.1[0.53, 2.28]	0.7931	-	
Level of education				
Less than high school	1[reference]		-	
More than high school	1.04[0.43, 2.53]	0.9287	-	
Drinking			-	
Never	1[reference]		1[reference]	
Occasional drink	1.86[0.86, 4]	0.114	1.98[0.97, 4.05]	0.0606
Frequent drink	3.16[1.01, 9.86]	0.0477	2.99[1.03, 8.66]	0.0434

Smoking status				
Never smoker	1[reference]		1[reference]	
Past smoker	2.71[1.15, 6.38]	0.0222	2.26[1.03, 4.94]	0.0421
Current smoker	5.75[2.18, 15.21]	0.0004	4.35[1.82, 10.38]	0.0009
Hypertension				
NO	1[reference]		1[reference]	
YES	1.91[0.8, 4.56]	0.1438	1.57[0.7, 3.53]	0.2754
Diabetes mellitus				
NO	1[reference]		-	
YES	0.98[0.46, 2.08]	0.9603	-	
Heart failure				
NO	1[reference]		-	
YES	0[0, Inf]	0.9852	-	
Stroke				
NO	1[reference]		-	
YES	1.14[0.32, 4.1]	0.8405	-	
Every taken prednisone or cortisone daily				
NO	1[reference]		-	
YES	2.05[0.6, 6.95]	0.2518	-	
Osteoporosis				
NO	1[reference]		1[reference]	
YES	3.2[1.36, 7.49]	0.0075	3.68[1.71, 7.89]	0.0008
History of Parents wrist fractures				
NO	1[reference]		1[reference]	
YES	0.22[0.05, 1.03]	0.0548	0.22[0.05, 1.02]	0.0528
Obesity				
NO	1[reference]		-	
YES	0.72[0.28, 1.84]	0.4913	-	
Chronic kidney diseases				
NO	1[reference]		-	
YES	1.62[0.18, 14.99]	0.6698	-	
Phosphate	2.95[1.73, 5.04]	0.0001	1[reference]	
Calcium	0.82[0.48, 1.41]	0.478	0.74[0.31, 1.78]	0.4962
